# A cross-sectional analysis of video games and attention deficit hyperactivity disorder symptoms in adolescents

**DOI:** 10.1186/1744-859X-5-16

**Published:** 2006-10-24

**Authors:** Philip A Chan, Terry Rabinowitz

**Affiliations:** 1 Department of Internal Medicine, Rhode Island Hospital, Brown University, Providence, RI, 02912, USA; 2 Department of Psychiatry, Fletcher Allen Healthcare, and the University of Vermont College of Medicine, Burlington, VT, 05401, USA

## Abstract

**Background:**

Excessive use of the Internet has been associated with attention deficit hyperactivity disorder (ADHD), but the relationship between video games and ADHD symptoms in adolescents is unknown.

**Method:**

A survey of adolescents and parents (n = 72 adolescents, 72 parents) was performed assessing daily time spent on the Internet, television, console video games, and Internet video games, and their association with academic and social functioning. Subjects were high school students in the ninth and tenth grade. Students were administered a modified Young's Internet Addiction Scale (YIAS) and asked questions about exercise, grades, work, and school detentions. Parents were asked to complete the Conners' Parent Rating Scale (CPRS) and answer questions regarding medical/psychiatric conditions in their child.

**Results:**

There was a significant association between time spent playing games for more than one hour a day and YIAS (p < 0.001), overall grade point average (p ≤ 0.019), and the "Inattention" and "ADHD" components of the CPRS (p ≤ 0.001 and p ≤ 0.020, respectively). No significant association was found between body mass index (BMI), exercise, number of detentions, or the "Oppositional" and "Hyperactivity" components of CPRS and video game use.

**Conclusion:**

Adolescents who play more than one hour of console or Internet video games may have more or more intense symptoms of ADHD or inattention than those who do not. Given the possible negative effects these conditions may have on scholastic performance, the added consequences of more time spent on video games may also place these individuals at increased risk for problems in school.

## Background

The introduction of the telegraph in the nineteenth century ushered in a new era of communication and social development. Further advances in technology led to the creation of the telephone, radio, and television. Recently, the Internet has become the pinnacle of interchange in the modern world and facilitates many different modes of communication. Each generation has raised concerns regarding the negative impact of media on social skills and personal relationships. The Internet appeals to adolescents for many reasons and has become a social connection for many with uses including messaging, e-mail, gaming, education, and music.

The Internet and other media types are reported to have important social and mental health effects in adolescents. The association between television viewing and obesity, attention disorders, school performance, and violence has been reported [1-6]. Likewise, recent studies on obsessive Internet use called "Internet Addiction" have shown negative effects on social health [7,8]. A significant relationship between Internet use and attention deficit hyperactivity disorder (ADHD) has also been shown in elementary school children [9]. Other studies have reported the similarities between computer video game addiction and pathological gambling or substance dependence [10-12].

The effect of video games on adolescents is not well characterized despite a growing body of evidence demonstrating their addictive nature and popularity [13-15]. Indeed, video game use may exceed that of television use in children [16]. In pre-adolescent teenagers, obesity has been linked to increasing time spent on video games, but other studies have disputed this finding in different populations [17-19]. Most studies of mental health and media use did not specifically examine video games, but included them as a subset of television or Internet use. One extensively studied area is the content of video games and their relationship to subsequent aggressive behavior in children [14,20-22]. Other case reports have documented associations between video games and various conditions such as epilepsy, musculoskeletal disorders, and deep vein thrombosis, although the strength of these associations has not yet been established [23-27].

Despite recent negative attention, some studies have shown possible positive effects of video games on development. One study by Li et al. found a positive association between motor development and cognitive behavior in preschool age children [28]. Other studies have reported that previous computer game experience enhances laparoscopic simulator performance in physicians [29]. In addition, video games are more frequently being used as adjuncts to learning and training in various settings, including medical education [30,31].

The term "video games" does not always differentiate between console and Internet/computer video games but instead, suggests a loose clustering. Console video games include Nintendo, Sony Playstation, Microsoft Xbox, and others. Internet video games refer to computer games played online in a community setting with other players. Although similar in nature, several important differences exist. Console games can be played with other people, but most games are "single player" and are meant to be played alone. Internet games however, are designed for "multi-player" use and are played with others online, usually at distant sites. Console games are less expensive than Internet games, and do not require a computer. The genre of video games played on the Internet versus console games also differs in content. Console game themes include sports, action, strategy, family, puzzle, role-playing games, and simulation, while video game themes designed for Internet use are more specific and are mainly action and strategy. The video game market, regardless of type, is a multi-billion dollar industry that generally targets children and adolescents.

The relationship between video games and ADHD is unknown. The incidence of ADHD continues to rise and is a significant challenge on medical, financial, and educational resources [32,33]. ADHD is a complex disorder that often requires input from the affected child or adolescent, teachers, parents, and physicians in order to be diagnosed correctly and treated successfully [34]. The Conners' Parent Rating Scale (CPRS) [35] is the most widely used instrument to aid in the diagnosis of children with ADHD. The CRS comprises both a parent and teacher questionnaire, and includes a number of components including oppositional behavior, hyperactivity, inattention, and ADHD.

This study examined the relationship between video game use and symptoms of ADHD. Other parameters studied included body mass index (BMI), school grades, work, detentions, and family situation.

## Method

### Design and procedures

After receiving IRB approval, subjects were recruited from a local high school in Vermont. Permission from school officials was obtained and contact was made with the guidance office and school teachers. Surveys were distributed to all 9^th ^and 10^th ^grade students at the school (n = 221). The survey included sections for students (five pages) and parents (two pages) to complete independently, as well as a consent form which needed to be signed by both the student and parent for participation in the study. All survey data was anonymous. Surveys were collected (n = 162) through the school Guidance Office. Eighteen surveys were omitted due to incomplete responses. The final subject pool comprised 144; 72 each from parents and students. Original power calculations were based on a reported 10% prevalence of psychiatric disorders in the adolescent population and called for a total of 200 students for a power of 0.80. However, statistically significant results were reached after analysis of 144 completed questionnaires and led us to conclude that the study could be terminated at that point.

### Measures

Time spent playing videos games, watching television, or using the Internet was assessed using a time scale of less than one hour, one to two hours, three to four hours, or greater than four hours. The student survey material included Young's Internet Addiction Scale, modified for video game use (YIAS-VG; internal consistency, alpha = 0.82) [36]. This scale was validated in previous studies for Internet addictive qualities [13,36]. The questions reflect the negative impact of video games on social functioning and relationships including excessive video game use, neglecting work and social life, anticipation, lack of control, and salience. Parents were surveyed using the Conners' Parent Rating Scale (CPRS; internal consistency, r = 0.57) [35]. The CPRS divides behavior into four categories: oppositional, hyperactivity, inattention, and ADHD. Other items included gender, family situation, exercise per week, detentions in the last month, work, and academic performance. Family situation was defined as either living with married parents or living with one parent who was divorced or separated. Academic performance was assessed by overall grade point average and last grade earned in both mathematics and English classes given that these two areas are accepted as core competencies in any high school curriculum.

### Data analysis

The dependent variables reported in numerical format (BMI, grades, YIAS-VG, CPRS) were analyzed using the student's t-test and the Mann-Whitney test. The latter method is based on median values and is the preferred method when testing small sample sizes. Data reported as "yes/no" (sex, work, detentions, exercise, and family situation) was analyzed using the chi-square test. Results were considered significant if p ≤ 0.05. Time spent playing video games, watching television, and using the internet was the independent variable. The time intervals compared were for student who spent less than one hour or greater than one hour on a particular activity. The one hour cutoff was used because it gave a more even distribution of sample sizes between the two groups although other time intervals were also compared.

## Results

The study cohort comprised 72 students; 31 males and 41 females in the ninth and tenth grade. Average age was 15.3 ± 0.7 years. Subject demographics are shown in Table 1. Almost 32% of students worked and 89% had parents who were married. Ten students had at least one detention in the last month and two students were involved in a physical fight in the last year. Four students consumed alcohol and one student reported daily smoking. Two students reported a diagnosis of ADHD and four reported having depression and/or anxiety.

**Table 1:** Subject Demographics

**Population Characteristics**	**Males **(n = 31)	**Females **(n = 41)	**Total **(n = 72)
Students who work	26% (8)	37% (15)	32% (23)
Parents married	87% (27)	90% (37)	89% (64)
Divorced, Separated, other	13% (4)	10% (4)	11% (8)
Detention in the last month	19% (6)	10% (4)	14% (10)
Physical fight in the last year	3% (1)	2% (1)	3% (2)
Drink alcohol weekly	3% (1)	7% (3)	6% (4)
Smoke daily	0% (0)	2% (1)	1% (1)
Diagnosed ADD/ADHD	6% (2)	0% (0)	3% (2)
Diagnosed Depression/Anxiety	3% (1)	7% (3)	6% (4)

The mean BMI for adolescents who watched less than one hour of television per day was 20.28 ± 2.33 and 22.11 ± 4.01 for those who watched more than one hour of television (p = 0.017, Table 2). There was a trend towards a higher BMI for adolescents spending more than one hour playing video games, but these results were not significant. No association was found between BMI and time spent on the Internet.

**Table 2:** Body Mass Index

		**INTERNET**		**TELEVISION**		**CONSOLE VIDEO GAMES**		**INTERNET VIDEO GAMES**	
		**< 1 hour**	**≥ 1 hour**	**< 1 hour**	**≥ 1 hour**	**< 1 hour**	**≥ 1 hour**	**< 1 hour**	**≥ 1 hour**
**Body Mass Index**	Mean	22.09	21.01	**20.28**	**22.11**	21.06	23.77	21.31	22.62
		± 4.12	± 3.15	± 2.33	± 4.01	± 3.13	± 5.19	± 3.59	± 3.73
	p-value	0.23		**0.017**		0.12		0.35	

Students who played video games for more than one hour had significant increases in scores on the YIAS-VG (p < 0.001 for console and Internet video games, Table 3). Other activities were associated with a trend toward increased YIAS-VG, but were not significant.

**Table 3:** Behavioral Symptoms

		**INTERNET**		**TELEVISION**		**CONSOLE VIDEO GAMES**		**INTERNET VIDEO GAMES**	
		**< 1 hour**	**≥ 1 hour**	**< 1 hour**	**≥ 1 hour**	**< 1 hour**	**≥ 1 hour**	**< 1 hour**	**≥ 1 hour**
**Young's Addiction Scale**	Mean	12.80	14.50	**9.70**	**16.00**	**10.10**	**34.30**	**10.20**	**38.60**
		± 11.00	± 16.40	± 14.60	± 13.80	± 10.70	± 14.80	± 10.40	± 13.40
	p-value	0.804		**0.040**		**< 0.001**		**< 0.001**	
									
**Conner's Scale: Oppositional**	Mean	1.77	2.66	2.36	2.23	2.33	2.00	2.33	1.89
		± 2.36	± 2.73	± 2.08	± 2.85	± 2.69	± 2.05	± 2.72	± 1.45
	p-value	0.096		0.397		0.917		0.826	
									
**Conner's Scale: Inattention**	Mean	1.29	1.68	1.72	1.40	**1.00**	**4.36**	**1.16**	**4.00**
		± 2.67	± 2.51	± 2.62	± 2.57	± 1.60	± 4.59	± 2.25	± 3.39
	p-value	0.289		0.311		**0.001**		**< 0.001**	
									
**Conner's Scale: Hyperactivity**	Mean	1.19	1.49	1.40	1.34	1.39	1.18	1.44	0.78
		± 1.14	± 2.11	± 1.32	± 1.96	± 1.82	± 1.40	± 1.79	± 1.39
	p-value	0.901		0.397		0.800		0.142	
									
**Conner's Scale: ADHD**	Mean	3.32	4.88	4.64	3.98	**3.59**	**7.64**	**3.78**	**7.22**
		± 3.75	± 5.13	± 4.66	± 4.64	± 3.89	± 6.79	± 4.26	± 6.12
	p-value	0.115		0.343		**0.018**		**0.020**	

ADHD = Attention deficit hyperactivity disorder

There was a significant increase in inattentive (p ≤ 0.001 for both Internet and console video games) and ADHD (p = 0.018 and 0.020 for console and Internet games, respectively) behavior in those who played video games for more than one hour (Table 3). No significant association was found between the hyperactivity or oppositional components of the CPRS and video game use. No significant relationship was found in any of the four categories and Internet or television use.

There was a trend toward lower grades in students who surf the Internet and play video games for more than one hour, but these results were not significant (Table 4). However, significantly lower grades were found between students who play video games for more than one hour and overall grade point average (GPA, p = 0.019 and 0.009 for console and Internet games, respectively).

**Table 4:** Academic Performance

		**INTERNET**		**TELEVISION**		**CONSOLE VIDEO GAMES**		**INTERNET VIDEO GAMES**	
		**< 1 hour**	**≥ 1 hour**	**< 1 hour**	**≥ 1 hour**	**< 1 hour**	**≥ 1 hour**	**< 1 hour**	**≥ 1 hour**
**Last Grade in Math**	Mean	3.422	3.308	3.306	3.383	3.393	3.132	3.37	3.259
		± 0.689	± 0.748	± 0.687	± 0.744	± 0.706	± 0.805	± 0.705	± 0.863
	p-value	0.547		0.422		0.255		0.754	
									
**Last Grade in English**	Mean	3.263	3.201	3.217	3.232	3.254	3.067	3.267	2.963
		± 0.721	± 0.662	± 0.608	± 0.723	± 0.659	± 0.829	± 0.638	± 0.934
	p-value	0.545		0.727		0.545		0.395	
									
**Overall Grade Point Average**	Mean	3.686	3.523	3.626	3.567	**3.668**	**3.002**	**3.668**	**3.00**
		± 0.392	± 0.494	± 0.438	± 0.475	± 0.341	± 0.762	± 0.356	± 0.701
	p-value	0.190		0.735		**0.019**		**0.009**	

Males were significantly more likely than females to spend greater than one hour a day playing console or internet video games (p < 0.001 and p = 0.003, respectively). Twenty males reported playing video games for more than one hour a day versus only one female adolescent reported playing internet video games for more than one hour. There was no significant relationship between gender and time spent watching television or on the Internet. We also found no significant association between time spent on any media form and students who worked, had married parents, received more detentions per month, or exercised more frequently.

## Discussion

ADHD among children and adolescents has been attributed to both genetic and environmental factors[37]. Of the media influences, only excessive Internet use has been reported to be associated with ADHD. The diagnosis of ADHD relies on input from teachers, parents, and physicians. This study found an increase in ADHD and inattention symptoms in adolescents who play video games for more than one hour a day.

The prevalence of ADHD in adolescents is reported to be 4–7% [37,38]. This study found a prevalence of 8.3% based on a reported diagnosis by a parent. It was not possible to determine the actual diagnosis of ADHD based only on the raw scores of the CRPS. More or more severe symptoms of inattention and ADHD behavior were found in students who played video games for more than one hour, but further study is needed to more clearly understand the association between video games and ADHD. It is unclear whether playing video games for more than one hour leads to an increase in ADHD symptoms, or whether adolescents with ADHD symptoms spend more time on video games.

This study found no association between video games use and oppositional or aggressive behavior. Previous research has shown a positive correlation between violence in video games and aggressive behavior [4,14,20,21]. It is possible that video games only lead to this type of behavior in groups prone to violent behavior or in conjunction with other forms of violence in media. The power of this study was not designed to detect such differences, thus no inferences can be made.

The effect of television viewing on BMI has been reported in several studies [1,2,5,6]. We found a significant association between increased BMI and watching television for more than one hour. Playing video games for more than one hour was not associated with an increase in BMI. Previous studies found a significant relationship between BMI and video games in younger populations [18,19]. Our findings suggest this association may persist into early adolescence.

Time on the Internet was not associated with increased BMI; a trend towards decreasing BMI was found in adolescents who use the Internet for more than one hour. Our findings suggest that current recommendations to limit television and video game times for children should be followed [6].

Both console and Internet video games were associated with an increase in addiction scores as measured by YIAS-VG. The YIAS-VG assesses the degree to which video games negatively impact different social factors including daily activities, relationships, sleep, and daily thoughts. The increase in YIAS-VG scores imply that playing video games for more than one hour a day does have a negative impact on relationships and daily activity. We did not define a cutoff on the YIAS-VG to identify "excessive" video game use but the scores in our cohort were not high enough to be considered as evidence of "Internet Addiction" [13,36].

GPA was lower in those who played video games for more than one hour. Even though this study cohort had a relatively high overall GPA, the difference between an "A" (less than one hour of video games) versus a "B" (more than one hour of video games) is a significant change in grade. For students who are less academically proficient, this may be especially important. There was also a trend towards a lower GPA in students who watch television for more than one hour. Excessive television has been reported to be associated with poor school performance [6].

This investigation found that playing console and Internet video games for more than one hour a day has negative social and academic effects in adolescents. This association does not depend on being "addicted" to video games or playing for excessive time periods. Furthermore, there was no difference between playing video games on the Internet or on a console system. The intensive nature of video games is likely to cause this time dependent relationship between video games and behavior disorders, regardless of whether it is over the Internet or on a console system.

Several limitations of the study exist. This cross-sectional comparison of video games and ADHD does not allow for cause-effect relationships to be established. Therefore, it is impossible to say whether playing video games leads to an increase in ADHD symptoms, or if adolescents with more ADHD symptoms tend to spend longer times playing video games. Prospective studies to examine this relationship more closely are certainly justified. The subject cohort was also not representative of all groups. The large majority of students who responded to the survey were Caucasian, not involved with drugs or alcohol, had married parents, and did well in school. Thus, the association between video games and ADHD in other cohorts cannot be inferred. This study was designed to analyze adolescents who spent more than one hour of time playing video games. It would be interesting to examine the latter cohort in more detail to determine if there is a linear relationship between time spent playing video games and ADHD symptoms or academic performance, or if some other relationship exists among those who spend excessive time on these activities.

## Conclusion

To our knowledge, this is the first study to find an association between video game use and ADHD symptoms in adolescents. Assessment of ADHD risk factors often involves identification of home and academic environmental factors. Parental relationships, early childhood development factors (i.e. preterm delivery), and excessive Internet use are associated with ADHD later in life. Identification of these and other risk factors that contribute to ADHD will lead to prevention and earlier treatment strategies.

## Appendix A

**Table 5:** UNIVERSITY OF VERMONT STUDENT SURVEY (To be completed individually by student)

**Age:**	**Height:**	**Weight:**				
**Sex: (Circle one)**	Male	Female				
**Occupation:**	Year in School:	Type of work (if any):				
**Last grade in Math Class (as appears on last report card):**	A+ A A- B+ B B- C+ C C- D+ D D- F					
**Last grade in English Class (as appears on last report card):**	A+ A A- B+ B B- C+ C C- D+ D D- F					
	Overall average grade (as appears on last report card):					
**How often do you exercise in an average week? (Circle one)**	Every day	4–6 days a week	1–3 days a week	Less than one day a week		
**What is your family situation:**	Parents are married or live together	Parents live apart	Parents are divorced, I live with mom or dad	Live with legal guardian		
						
**Average total number of hours a day spent on Internet: (Circle one)**	Less than 1	1–2	3–4	More than 4		
**Average total number of hours a day spent on Computer games: (Circle one)**	Less than 1	1–2	3–4	More than 4		
**Average total number of hours a day spent on other console games such as Playstation, N64, etc. (Circle one)**	Less than 1	1–2	3–4	More than 4		
**Total number of hours a day spent watching television: (Circle one)**	Less than 1	1–2	3–4	More than 4		
**Type of games played : (Circle as many as apply)**	Sports	Strategy	Action	Other		
**Favorite game:**						
**How many detentions have you had in the past month?**						
**Have you been suspended from school in the past year?**	YES	NO	If yes, how many times?			
**Have you been in a physical fight in the past year?**	YES	NO	If yes, how many times?			
**Have you been arrested in the past year?**	YES	NO	If yes, how many times?			
**Do you drink alcohol? (Circle one)**	YES	NO				
	If yes, how many drinks per week on average?					
**Do you smoke? (Circle one)**	YES	NO				
	If yes, how many cigarettes per day on average?					
**Do you smoke marijuana? (Circle one)**	YES	NO				
	If yes, how many joints per week on average?					
**Other drugs and amount:**						
**How often do you use alcohol/cigarettes/drugs while playing video games? (Circle one)**	ALWAYS	SOMETIMES	RARELY	NEVER		
						
	**Rarely**	**Occasionally**	**Frequently**	**Often**	**Always**	**Does Not Apply**
**1. How often do you find that you play video games longer than you intended?**						
**2. How often do you neglect household chores to spend more time playing video games?**						
**3. How often do you prefer the excitement of playing video games to spending time with your girl/boy friend or family?**						
**4. How often do you play video games with friends?**						
**5. How often do others in your life complain to you about the amount of time you spend playing video games?**						
**6. How often do your grades or schoolwork suffer because of the amount of time you spend playing video games?**						
**7. How often do you play video games before something else that you need to do?**						
**8. How often does your job performance or productivity suffer because of playing video games?**						
**9. How often do you become defensive or secretive when anyone asks you how much time you spend playing video games?**						
**10. How often do you block out disturbing thoughts about your life with thoughts of playing video games?**						
**11. How often do you find yourself anticipating when you will play video games again?**						
**12. How often do you fear that life without video games would be boring, empty, and joyless?**						
**13. How often do you snap, yell, or act annoyed if someone bothers you while you are playing video games?**						
**14. How often do you lose sleep due to late-night video games?**						
**15. How often do you feel preoccupied with playing video games when not, or fantasize about doing so?**						
**16. How often do you find yourself saying "just a few more minutes" when playing video games?**						
**17. How often do you try to cut down the amount of time you spend playing video games and fail?**						
**18. How often do you try to hide how long you've been playing video games?**						
**19. How often do you choose to spend more time playing video games over going out with others?**						
**20. How often do you feel depressed, moody, or nervous when you are not playing video games, which goes away once you are back playing?**						
						
Subjects were also asked to complete the Beck Depression Inventory.						
**Production note: This article was amended post-publication. For copyright reasons, the Beck Depression Inventory was removed.**						

Thank you for taking part in our survey. All results are confidential. Please contact Philip Chan (research coordinator) at Philip.Chan@uvm.edu anytime with questions.

PLEASE PLACE FINISHED SURVEY IN A SEALED ENVELOPE

## Appendix B

**Table 6:** UNIVERSITY OF VERMONT PARENT SURVEY (To be completed by the parent/guardian who is most involved in the student's care/daily life)

**Relation to student: (Circle one)**	Father	Mother	Legal Guardian	Other (specify):
**Number of hours child spends a day on Internet total: (Circle one)**	< 1	1–2	3–4	>4
**Number of hours a day child spends on computer games: (Circle one)**	< 1	1–2	3–4	>4
**Number hours a day child spends on console games (Playstation, Nintendo, etc.): (Circle one)**	< 1	1–2	3–4	>4
**Number hours a day child spends watching television: (Circle one)**	< 1	1–2	3–4	>4
**Has your son/daughter ever been diagnosed with: (Circle one)**	Depression	Anxiety Disorder	ADD/ADHD	Other (specify):
**If so, for how many years?**				
**Does your son/daughter have any other medical illnesses (diabetes, epilepsy, etc.)?**	Yes	No	If so, please specify:	
**Is your son/daughter currently on any prescription medications?**	Yes	No		
**If so, please list medications:**				
**Is your son/daughter:**	**NOT TRUE AT ALL (NEVER, SELDOM)**	**JUST A LITTLE TRUE (SOMETIMES)**	**PRETTY MUCH TRUE (OFTEN)**	**VERY MUCH TRUE (VERY OFTEN)**
**1 Inattentive, easily distracted**				
**2 Angry and resentful**				
**3 Difficulty doing or completing homework**				
**4 Always "on the go"**				
**5 Short attention span**				
**6 Argues with adults**				
**7 Fidgets with hands/feet, squirm in seat**				
**8 Fails to complete assignments on time**				
**9 Hard to control in malls or grocery shopping**				
**10 Messy or disorganized at home or school**				
**11 Loses temper**				
**12 Needs close supervision to get through assignments**				
**13 Only attends if it is something he/she is very interested in**				
**14 Runs or climbs excessively in situations where it is inappropriate**				
**15 Distractibility or attention span a problem**				
**16 Irritable**				
**17 Avoids, expresses reluctance about, or has difficulty engaging in tasks that require sustained mental effort (such as school work or homework)**				
**18 Restless in the "squirmy" sense**				
**19 Gets distracted when given instruction to do something**				
**20 Actively defies or refuses to comply with adults' requests**				
**21 Has trouble concentrating in class**				
**22 has difficulty waiting in lines or awaiting turn in games or group situations**				
**23 Leaves seat in classroom or other situation in which remaining seated is expected**				
**24 Deliberately does things that annoy other people**				
**25 Does not follow through on instructions and fails to finish schoolwork, chores, or duties in the workplace**				
**26 has difficulty playing or engaging in leisure activities quietly**				
**27 Easily frustrated in efforts**				

Thank you for taking part in our survey. All results are confidential. Please contact Philip Chan (research coordinator) at Philip.Chan@uvm.edu anytime with questions.

PLEASE PLACE FINISHED SURVEY IN A SEALED ENVELOPE

## Production note

This article was amended post-publication. The Beck Depression Inventory was originally listed in Appendix A (Table 5), but has been removed for copyright reasons.
